# Intracellular ATP Concentration Contributes to the Cytotoxic and Cytoprotective Effects of Adenosine

**DOI:** 10.1371/journal.pone.0076731

**Published:** 2013-10-03

**Authors:** Shujue Li, Xiaofen Li, Haiping Guo, Shouting Liu, Hongbiao Huang, Ningning Liu, Changshan Yang, Ping Tang, Jinbao Liu

**Affiliations:** 1 Protein Modification and Degradation Lab, Department of Pathophysiology, Guangzhou Medical University, Guangzhou, Guangdong, People's Republic of China; 2 Department of Urology, the First Affiliated Hospital, Guangzhou Medical University, Guangzhou, Guangdong, People's Republic of China; 3 Guangzhou Research Institute of Cardiovascular Disease, the Second Affiliated Hospital, Guangzhou Medical University, Guangzhou, Guangdong, People's Republic of China; Sun Yat-sen University Cancer Center, China

## Abstract

Extracellular adenosine (Ade) interacts with cells by two pathways: by activating cell surface receptors at nanomolar/micromolar concentrations; and by interfering with the homeostasis of the intracellular nucleotide pool at millimolar concentrations. Ade shows both cytotoxic and cytoprotective effects; however, the underlying mechanisms remain unclear. In the present study, the effects of adenosine-mediated ATP on cell viability were investigated. Adenosine treatment was found to be cytoprotective in the low intracellular ATP state, but cytotoxic under the normal ATP state. Adenosine-mediated cytotoxicity and cytoprotection rely on adenosine-derived ATP formation, but not via the adenosine receptor pathway. Ade enhanced proteasome inhibition-induced cell death mediated by ATP generation. These data provide a new pathway by which adenosine exerts dual biological effects on cell viability, suggesting an important role for adenosine as an ATP precursor besides the adenosine receptor pathway.

## Introduction

Extracellular adenosine (Ade) interacts with cells by two pathways: by activating cell surface receptors at nanomolar/micromolar concentrations; and by interfering with the homeostasis of the intracellular nucleotide pool at millimolar concentrations [1]. Studies have reported that Ade shows contradictory effects: on the one hand, Ade impairs cell proliferation [2]–[4], and induces apoptosis and cell death [5], [6]; on the other hand, Ade provides cytoprotective functions in the heart and brain during ischemia, hypoxia, or ischemia-reperfusion [7]–[9]. However, the mechanism of Ade-mediated cytotoxic and cytoprotective effects remains unclear.

A number of studies have shown that cell-specific purinergic receptors contribute to the dual effects of Ade on cell viability [10]–[13]. Ade is a key endogenous molecule that regulates tissue function by activating four G-protein-coupled Ade receptors: A_1_, A_2A_, A_2B_, and A_3_ [14]. Besides the receptor pathway, Ade also has an important role in biochemical processes, such as energy transfer – ATP and ADP – as well as in signal transduction as cAMP. To date, many conflicting studies have found it difficult to explain the cytotoxic or cytoprotective effects of Ade using the Ade receptor pathway hypothesis.

Previously, we reported that intracellular ATP at physiological levels bidirectionally regulates 26S proteasome proteolytic function in the cell [15]. Since the ubiquitin-proteasome system (UPS), by regulating protein balance, has an important role in multiple cellular processes, including cell viability and cell death, we hypothesized that Ade-mediated ATP could possibly contribute to the cytotoxic and cytoprotective effects of Ade. Here, we report that intracellular ATP concentration determines the cytotoxic or cytoprotective effects of Ade on the cell, suggesting a novel pathway besides the Ade receptor pathway.

## Materials and Methods

### Materials

Adenosine, dipyridamole (DP), and 8-SPT were obtained from Sigma-Aldrich Inc. (St. Louis, MO, USA). Rabbit polyclonal antibodies against GAPDH (FL-335) and nuclear poly (ADP-ribose) polymerase (PARP) were from Santa Cruz Biotechnology Inc. (Santa Cruz, CA, USA) and Cell Signaling (Beverly, MA, USA) respectively. All cell lines were originally purchased from the American Type Culture Collection (ATCC). Mouse thymocytes were prepared as previously reported [16].

### ATP content determination

ATP content determination was performed as described previously [10]. Briefly, equal numbers of cultured cells were collected, and the cell pellet was immediately frozen and stored in liquid nitrogen for subsequent ATP analysis. The lysates were centrifuged at 12,000 rpm for 10 min at 4°C. The supernatant was collected for analyzing ATP using a reversed-phase C18 HPLC assay (LC-6AD; Shimadzu, Japan) and the pH was adjusted to 7.4. The mobile consisted of 180 mM of KH_2_PO_4_ (5% methanol) (pH 6.25) running at 0.8 ml/min. The assay was linear from 0.05 to 200 µg/mL for ATP with a coefficient of determination (R^2^) >0.999. Validation coefficients of variation for intra- and inter-day assays were less than 1.5% and 5.1% respectively. Relative ATP content was calculated according to the peak area versus the ATP standard curve [15]. ATP assay was performed in independent repeated experiments as indicated.

### Western blot analysis

Western blot was performed as we described previously [17]. Briefly, an equal amount of total protein extracted from cultured cells were separated by 12% SDS-PAGE and transferred to polyvinylidene difluoride (PVDF) membranes. The blots were blocked with 5% milk for 1 h. Primary Abs and horseradish peroxidase (HRP)-conjugated secondary Abs were each incubated for 1 h. The bounded secondary antibodies were reacted to the ECL detection reagents and exposed to X-ray films (Kodak, Japan).

### Cell viability assay

The effects of drugs on the cell viability were determined using the MTS assay (CellTiter 96® AQueous One Solution Cell Proliferation Assay; Promega Corporation, Madison, WI, USA) as reported previously [9]. Briefly, cells were cultured in 96-well plates and treated with the indicated agents for 24 or 48 h. Treated cells were then incubated with 20 µL of MTS for an additional 3 h. The absorbance was measured at 490 nm using an automatic microplate reader (Sunrise, Tecan Group Ltd., Switzerland). Cell viability was calculated using the following formula: cell viability (%)  =  [(average absorbance of treated group – average absorbance of blank)/(average absorbance of untreated group – average absorbance of blank)] ×100% [15]. MTS assay was performed in replicated experiments.

### Cell death assay

Apoptosis assay was performed as we previously described [17]. In brief, cultured cells were harvested and washed with cold PBS and resuspended with the binding buffer, followed by Annexin V- FITC incubation for 15 min and PI staining for another 15 min at 4°C n dark. The stained cells were analyzed with flow cytometry within 30 min. Cell death includes all the non-viable cells (PI/Annexin V-negative).

The morphological changes of cell death were performed as we reported [17]. To monitor temporal changes in the incidence of cell death in the live culture condition, cells were seeded into 12-well plates and PI was added directly to the cell culture medium, then the cells in the culture dish were kinetically imaged with an inverted fluorescence microscope equipped with a digital camera (Axio Obsever Z1, Zeiss).

### Statistical methods

Mean ±SD are presented where applicable. Unpaired Student's t-test or one- way ANOVA is used for determining statistical probabilities. P values of less than 0.05 were considered significant.

## Results

### Ade caused increases of intracellular ATP content

**Figure 1:**
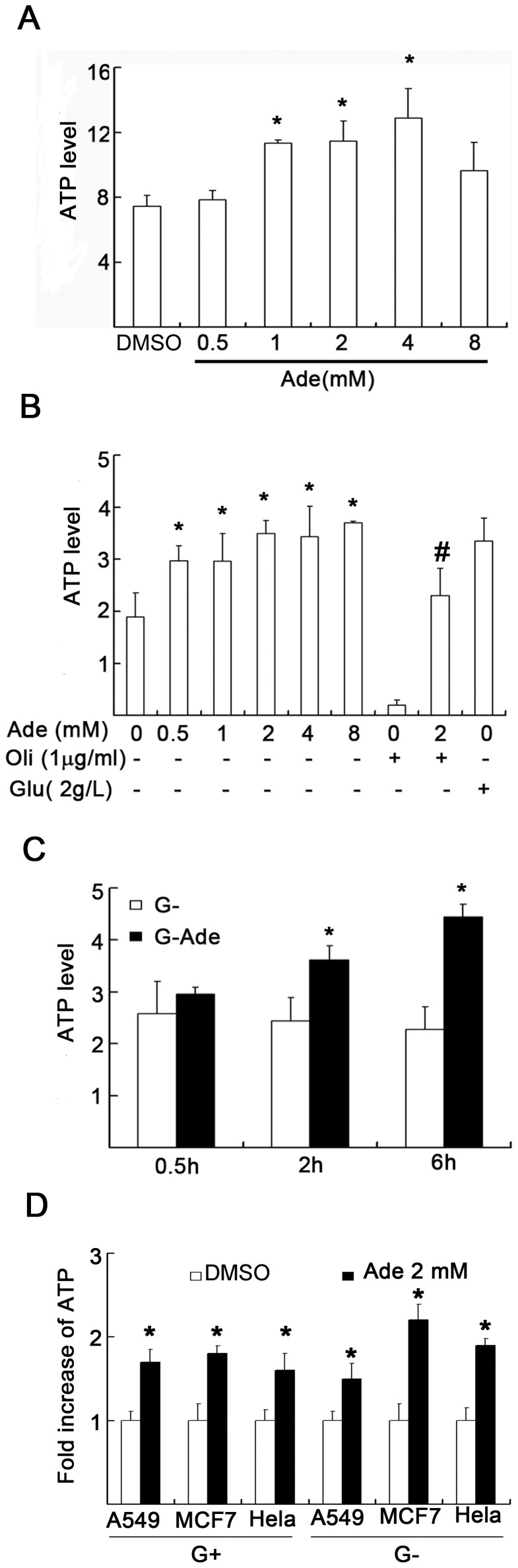
Adenosine (Ade) increases intracellular ATP contents in multiple cells. (A) Primary thymocytes were exposed to either vehicle DMSO (DM) or Ade in normal culture medium for 4 h and cells were collected for total ATP assay by HPLC (LC-6AD; Shimadzu). ATP contents (µM) of equal number of cells (2×10^5^) were compared (n = 4). Each column represents the average of independent repeated experiments. Mean ±SD. *p<0.05 compared to controls. (B) K562 cells were treated with indicated doses of Ade or oligomycin (Oli; 1 µg/ml) in the absence of d-glucose in RPMI 1640 medium for 6 h. d-glucose (2 g/L) was used as a positive control. Mean ±SD (n = 4). *p<0.05 vs. control; ^#^p<0.05 vs. Oli treatment alone. (C) K562 was exposed to 2 mM of Ade for 0.5, 2, and 6 h in the absence of d-glucose in the culture medium. Mean ±SD. *p<0.05 vs. 0.5 h treatment. (D) Increase of ATP in multiple cell lines: A549, MCF7, and Hela cells were exposed to either DMSO or 2 mM of Ade for 6 h in the absence (G-) or presence of d-glucose (2 g/L, G+) in the culture medium. ATP contents were detected by HPLC (n = 4) and the increase of ATP after Ade treatment was calculated as: Ade-treated/vehicle-treated. All controls were set as 1.0.

To detect intracellular ATP levels, primary lymphocytes and multiple cancer cell lines were incubated with millimolar levels of Ade, and ATP content was detected by HPLC. First, we observed the effect of Ade on intracellular ATP content both in primary lymphocytes and in K562 cell lines. We found that in primary cultured thymocytes, Ade increased ATP content to 5%, 52%, 54%, 72%, and 30% at 0.5, 1, 2, 4, and 8 mM levels, respectively, in the presence of d-glucose (Fig. 1A). In K562 cancer cells, Ade increased ATP content to 57%, 57%, 85%, 82%, and 96% at 0.5, 1, 2, 4, and 8 mM levels, respectively, and oligomycin (Oli), an F_o_F_1_-ATPase inhibitor, almost abolished ATP production, but did not affect Ade-mediated ATP production in the absence of d-glucose in the culture medium (Fig. 1B). Secondly, kinetic changes in ATP in the cells cultured in the d-glucose-free medium treated with 2 mM Ade were detected. Ade kinetically increased intracellular ATP contents (Fig. 1C). We further compared the ATP difference with Ade treatment (2 mM) in cells cultured in the absence or presence of d-glucose in the medium along with other cell lines, including A549, MCF-7, and Hela cells. The results showed that all cells showed increased ATP contents (Fig. 1D), either in the absence or presence of d-glucose in the culture medium. These results demonstrated that Ade can efficiently increase intracellular ATP contents in all the detected cells.

### Ade decreases cell viability and induces cell death in normal ATP states

**Figure 2:**
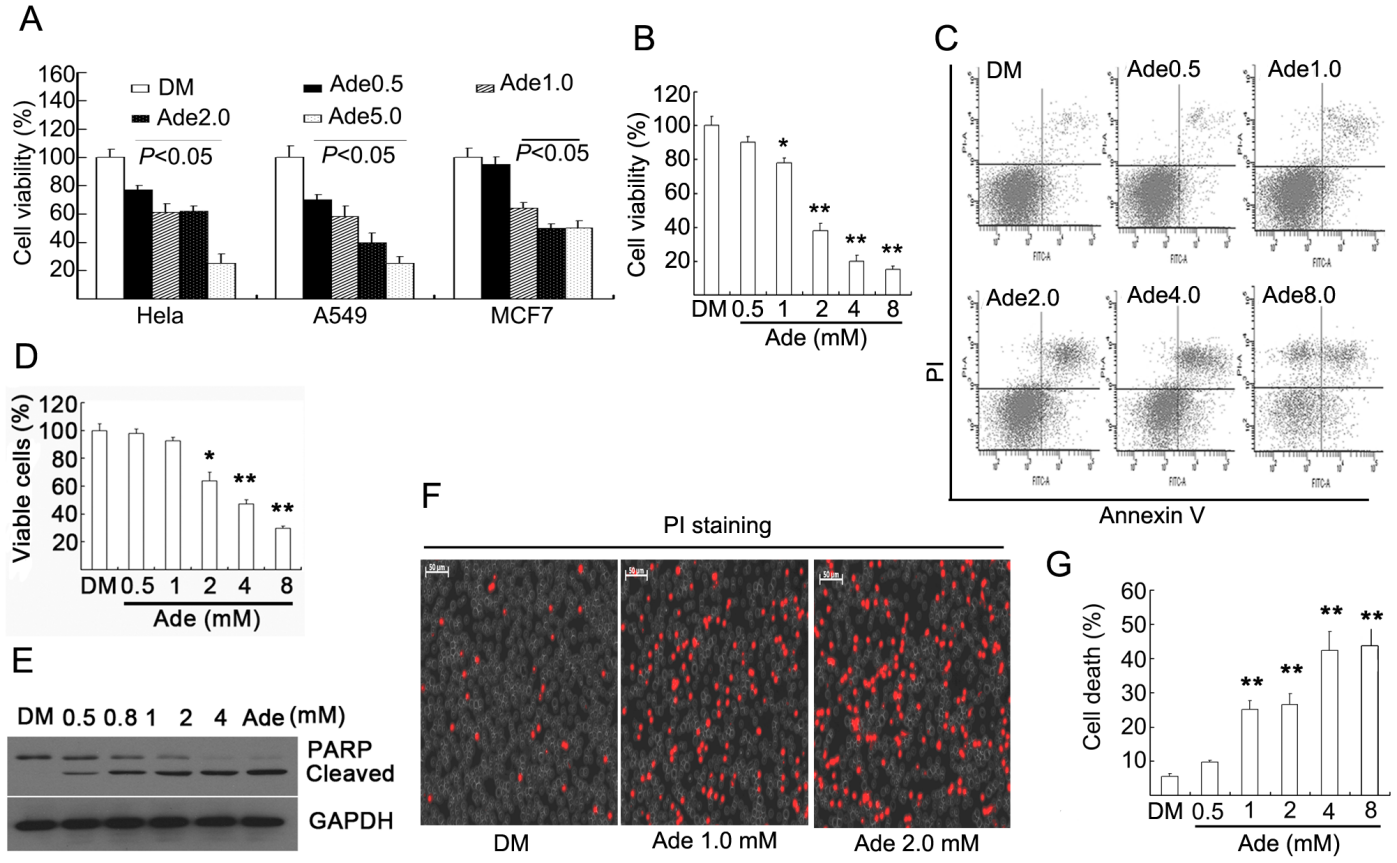
Ade decreases cell viability and induces cell death. (A) A549, MCF7, and Hela cells were exposed to Ade as indicated in normal culture medium for 72 h. Cell viability was detected using the MTS assay. Each column represents the average of five replicated experiements. Mean ±SD (n = 5). p<0.05, vs. vehicle control. (B) Ana-1 cells were treated with Ade for 72 h, cell viability was detected as in (A). Mean ±SD (n = 5). *p<0.05, **p<0.01, vs. vehicle control. (C, D, E) Ana-1 cells were incubated with Ade in normal culture medium for 12 h, then cell apoptosis was detected by either flow cytometry (FACScan; BD Biosciences) or Western blot. Representative cell death image and cell death data in Ana-1 cells are shown in (C, D). Mean ±SD (n = 3). *p<0.05, **p<0.01 vs. vehicle control. PARP cleavage is shown in (E). GAPDH was used as a loading control. (F, G) Thymus lymphocytes were incubated with Ade as indicated for 12 h, cell death was detected. Cell death images by PI staining in living cells under an inverted fluorescence microscope were shown in (F) and cell death data by flow cytometry are summarized in (G). **p<0.01 *vs.* vehicle control. Mean ±SD (n = 3).

Using the MTS assay, we found that Ade treatment (0.5, 1, 2, and 5 mM) for 72 h decreased cell viability in a dose-dependent manner in the detected three cell lines cultured in normal culture medium (Fig. 2A). In the detected cancer lines, Ade did not induce cell death via an unknown mechanism. Compared to cancer cells, Ade not only dose-dependently decreased cell viability (Fig. 2B) but also induced cell death in murine macrophage cell line Ana-1 cells. Ade at levels of 2, 4, and 8 mM caused 40%, 50%, and 70% cell death, respectively, detected using Annexin V-PI double staining by flow cytometry (Figs 2C, D), which was further confirmed by detecting PARP cleavage indicative of apoptosis (Fig. 2E). In cultured primary lymphocytes, cell death was observed using PI staining in living thymocytes after 12 h Ade treatment (Fig. 2F). Ade also induced typical cell death in a dose-dependent manner by PI/Annexin V staining, and 2, 4 mM levels of Ade caused 25% and 45% cell death respectively (Fig. 2G). These results demonstrated that Ade could decrease cell viability in normal culture conditions.

### Ade increases cell viability in low ATP states and rescues ATP-depletion-induced cell death

**Figure 3:**
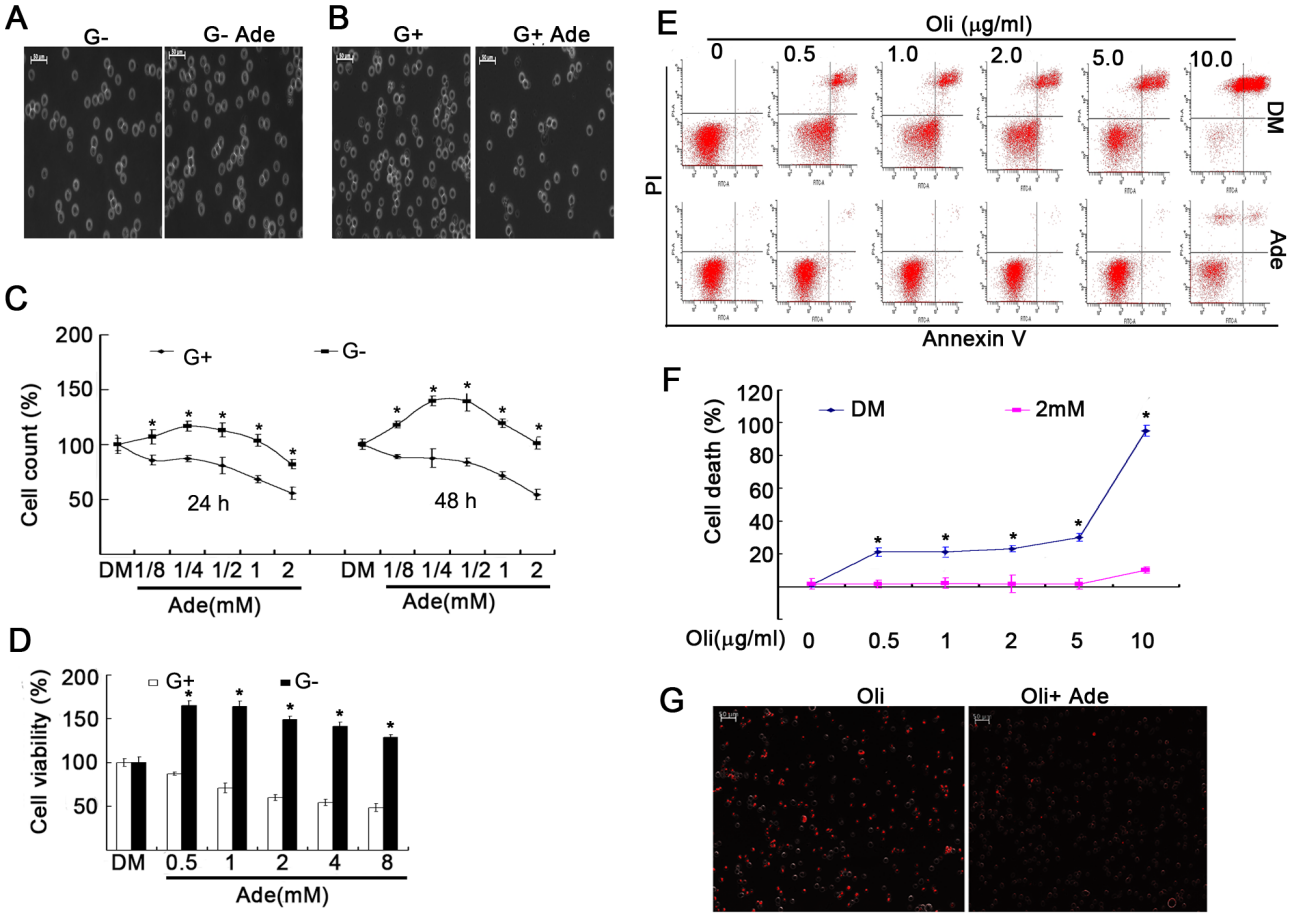
Ade increases cell viability in low ATP states and rescued cell death induced by ATP-depletion. (A, B) K562 cells were exposed to 2 mM of Ade cultured in RPMI 1640 medium with or without d-glucose for different time points. Cell density was imaged using an inverted microscope (Axio Obsever Z1; Zeiss, Germany). Typical images of cell density were selected from cells treated with Ade in d-glucose-free medium for 24 h (A) or in d-glucose-containing medium for 72 h (B). Scale bar  = 50 µm. (C) K562 cells were exposed to Ade for 24 h (left) or 48 h (right) with or without d-glucose; absolute cell numbers were counted using a cell counter. Mean ±SD (n = 3). *p<0.05 vs. d-glucose-containing cells. (D) K562 cells were incubated with Ade with or without d-glucose for 36 h. Cell viability was detected using the MTS assay. Mean ±SD (n = 3). *p<0.05 *vs.* glucose-containing cells. (E, F) K562 cells were treated with Oli with or without Ade (2 mM) in the d-glucose-free RPMI 1640 medium for 6 h, then cell apoptosis was detected by flow cytometry. Typical flow images are shown in (E) and cell death in (F). Mean ±SD (n = 3). *p<0.05 vs. Ade-treated cells. (G) K562 cells were treated with Oli (1 µg/ml) and Ade (2 mM) for 18 h in the glucose-free medium, and cells were then stained with PI and dynamically recorded under an inverted epi-fluorescent microscope. A typical image is shown. Scale bar  = 50 µM.

To set a low ATP state, d-glucose was withdrawn from the culture medium [15]. In d-glucose-free medium, cells depend on a non-glucose pathway for their ATP supply. Under these conditions, Ade efficiently increased ATP content (Fig. 1). Cell density was directly observed under a phase-contrast microscope and absolute cell numbers were count. Ade increased cell density and living cells in K562 cells cultured in d-glucose-free medium, while in K562 cells cultured with d-glucose-containing medium, Ade dramatically inhibited cell density (Figs 3A–C). Using the alamurBlue Assay, it was confirmed that Ade inhibited cell viability with d-glucose in the culture medium, while in cells cultured in d-glucose-free medium, Ade increased cell viability, and the highest cell viability occurred at relatively low levels of Ade (0.5 and 1.0 mM) (Fig. 3D).

To mimic the ATP depletion state, Oli was added to the d-glucose-free culture medium to block oxidative phosphorylation [15], [18], [19]. In K562 cells cultured in d-glucose-free medium, Oli treatment dose-dependently induced cell death and abolished intracellular ATP, both of which were rescued in the presence of 2 mM Ade (Fig. 1B and Figs 3E–G). These results indicated that Ade increased cell viability in low ATP states.

### DP, but not 8-SPT, affects Ade-mediated cytoprotective or cytotoxic effects

**Figure 4:**
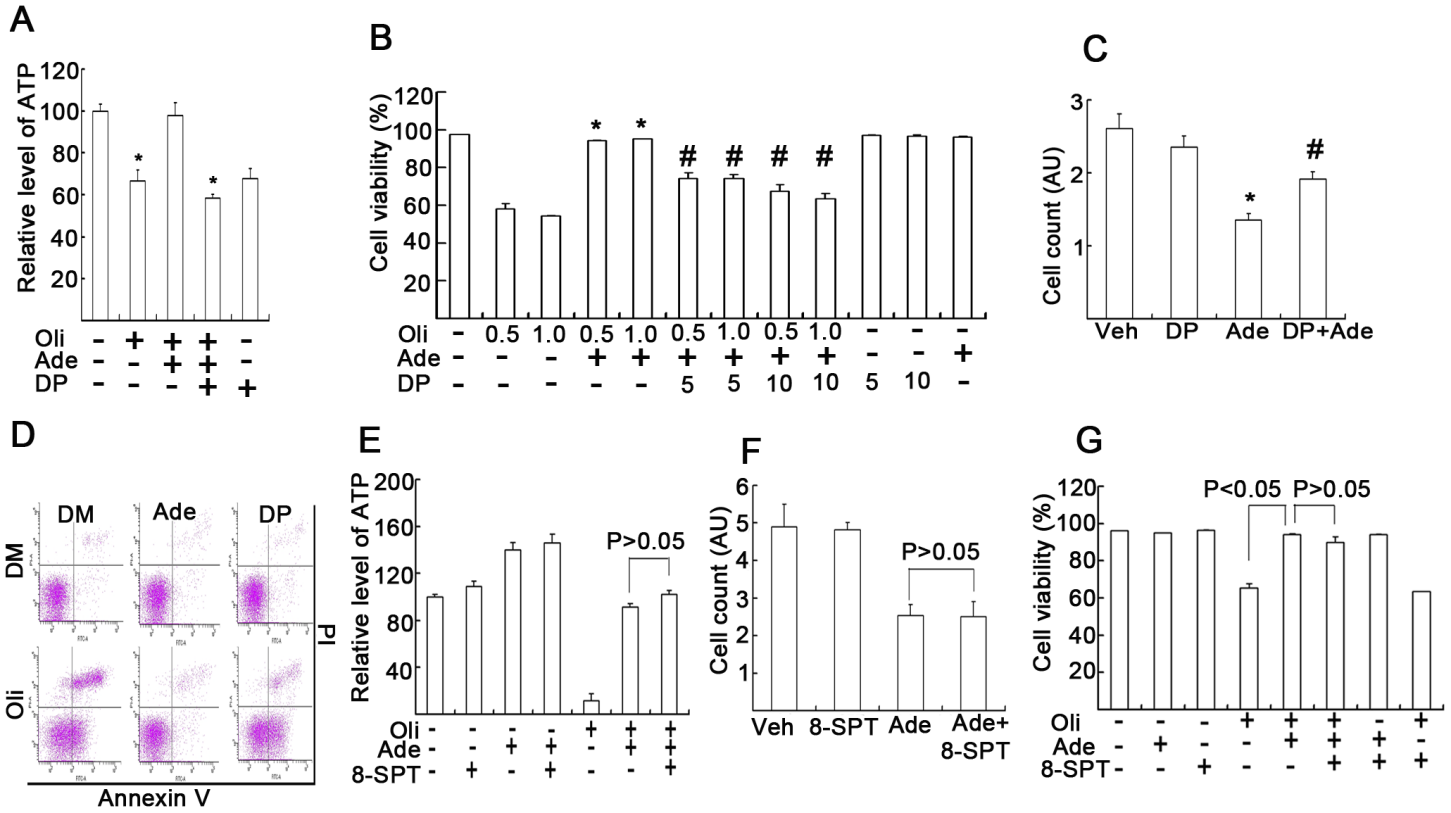
Ade transportation, but not Ade receptors, is required for Ade to exert its cytotoxic or cytoprotective effects. (A) K562 cells cultured in glucose-free culture medium were treated with Oli (0.5 µg/ml), Ade (2 mM) and DP (10 µM) for 3 h, followed by HPLC ATP assay. Mean ±SD (n = 3). *p<0.05 vs. Oli+Ade treatment. (B) K562 cells were cultured and treated in the d-glucose-free medium as indicated for 18 h, and cells were then stained with Annexin V/PI followed by flow cytometry. Viable cells are shown. Mean ±SD (n = 3). *p<0.05 each compared with Oli treatment alone; #p<0.05 each compared with Oli+Ade combination treatment. (C) K562 cells were cultured in normal medium and exposed to 2 mM Ade and DP (10 µM) for 18 h, cell numbers were counted using a cell counter. Mean ±SD (n = 3). *p<0.05 compared with vehicle control; ^#^p<0.05 compared with Ade treatment alone. (D) As treated in (B), typical flow images are shown (Ade: 2 mM, Oli: 1.0 µg/ml, DP: 10 µM). (E) K562 cells were cultured in d-glucose-free medium and treated with the agents as indicated (Oli: 1.0 µg/ml, Ade: 2 mM, 8-SPT: 10 µM) for 4 h followed by ATP assay. Mean ±SD (n = 3). (F) K562 cells were cultured in normal glucose-containing medium and treated as indicated (Ade: 2 mM, 8-SPT: 10 µM) for 18 h, cell numbers were counted and summarized. Mean ±SD (n = 3). (G) K562 cells were cultured in glucose-free medium and treated as indicated (Oli: 1.0 µg/ml, 8-SPT: 10 µM) for 15 h, cell death was detected by flow cytometry. Mean ±SD (n = 3).

To block Ade entry to the cell, DP, an inhibitor of the Ade transmembrane carrier, was used to observe its effects on intracellular ATP concentrations and cell viability [20], [21]. In the absence of d-glucose in the culture medium, Oli (0.5 µg/ml) depleted 40% intracellular ATP after 3 h treatment (Fig. 4A) and depleted more than 90% intracellular ATP after 6 h treatment (Fig. 1B). With the addition of Ade (2 mM), ATP increased to normal levels and DP (10 µM) significantly reversed the Ade-mediated increase in ATP (Fig. 4A). It was further shown that ATP depletion by Oli (0.5 and 1 µg/ml) induced 40%, 50% cell death, respectively, while with the addition of Ade, cell death was almost completely rescued and pretreatment with DP reversibly increased cell death (Figs 4B, D). To further observe the effect of DP on Ade-mediated inhibition on cell viability in normal states, cells were exposed to both Ade and DP for 18 h. It was found that 2 mM of Ade decreased cell viability, whereas DP clearly reversed Ade-mediated effect (Fig. 4C). These data showed that Ade-mediated cytotoxicity and cytoprotection were dependent on Ade regulation of intracellular ATP concentrations.

To further investigate whether the Ade receptor pathway is associated with the cytotoxic and cytoprotective effects of Ade under these conditions, 8-SPT, a non-selective Ade receptor antagonist, was used [22]. K562 cells were treated with 8-SPT in the absence or presence of Ade or Oli; cell numbers were count and cell death was detected by flow cytometry. We found that 8-SPT did not significantly affect the Ade-mediated ATP increase (Fig. 4E). Oli treatment alone induced both cell proliferation arrest and cell death in the absence of d-glucose in the culture medium, and the presence of Ade mostly rescued Oli-induced changes, which were unaffected by the addition of 10 µM 8-SPT (Figs. 4F, G).

### Ade-derived ATP concentration determines the sensitivity to proteasome inhibition-induced cell death

**Figure 5:**
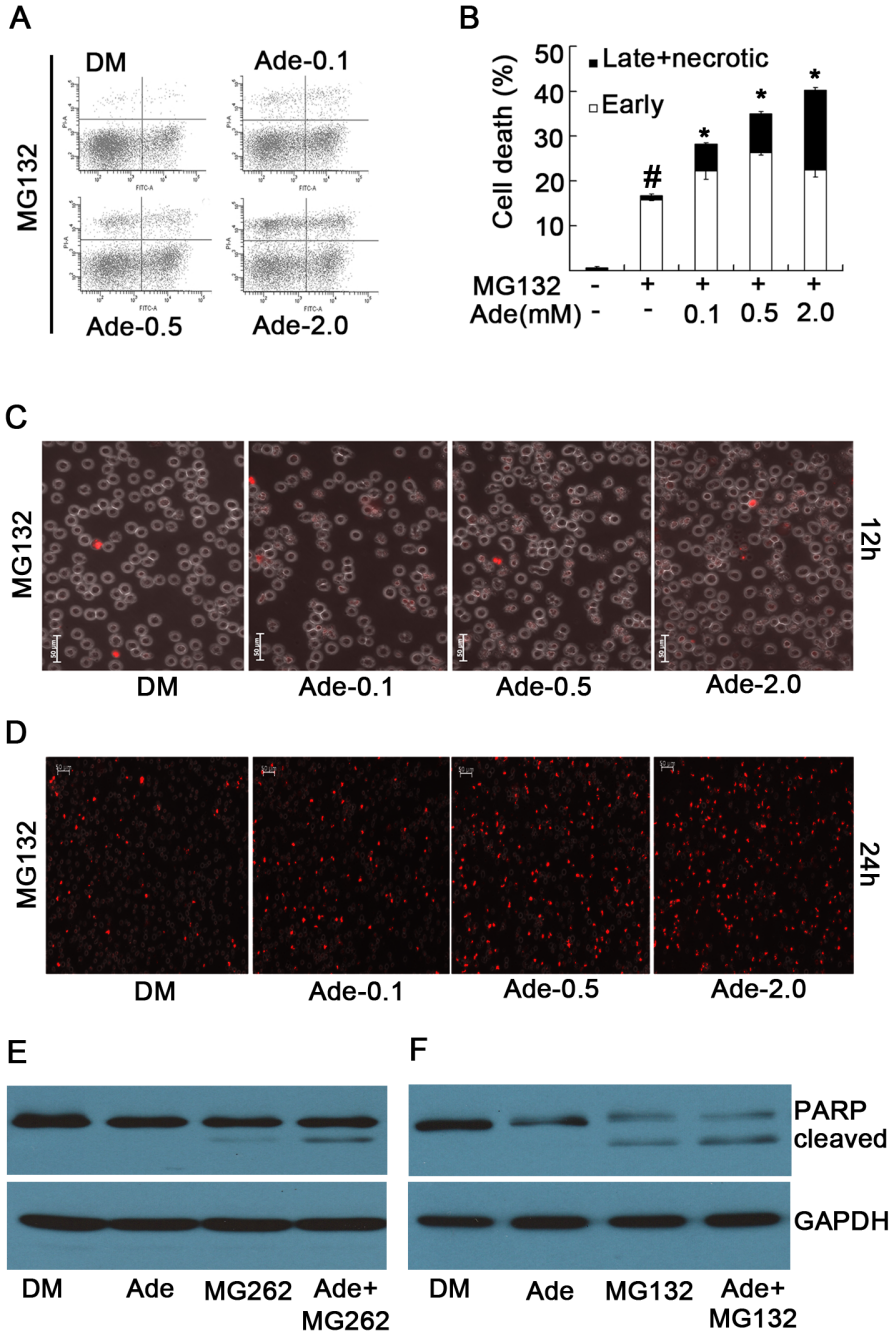
Ade increases the sensitivity to proteasome inhibition-induced cytotoxicity. (A,B) K562 cells were treated with proteasome inhibitor MG132 (5 µM) for 15 h in the presence of various doses of Ade; cell apoptosis was detected using Annexin V/PI staining by flow cytometry. Typical flow images are shown in (A) and the cell death data summarized in (B). Mean ±SD (n = 3).*p<0.05 vs. MG132 treatment alone; #p<0.05 vs. control. (C, D) As treated in (A) dynamically, cell death was detected with PI staining in live cells under an inverted microscope. Typical images at 12 h and 24 h are shown in (C) and (D) respectively. (E) K562 cells were treated with MG132 (5 µM), Ade (2 mM) and the combination for 12 h, PARP was detected by Western blot. GAPDH was used as a loading control.

**Figure 6:**
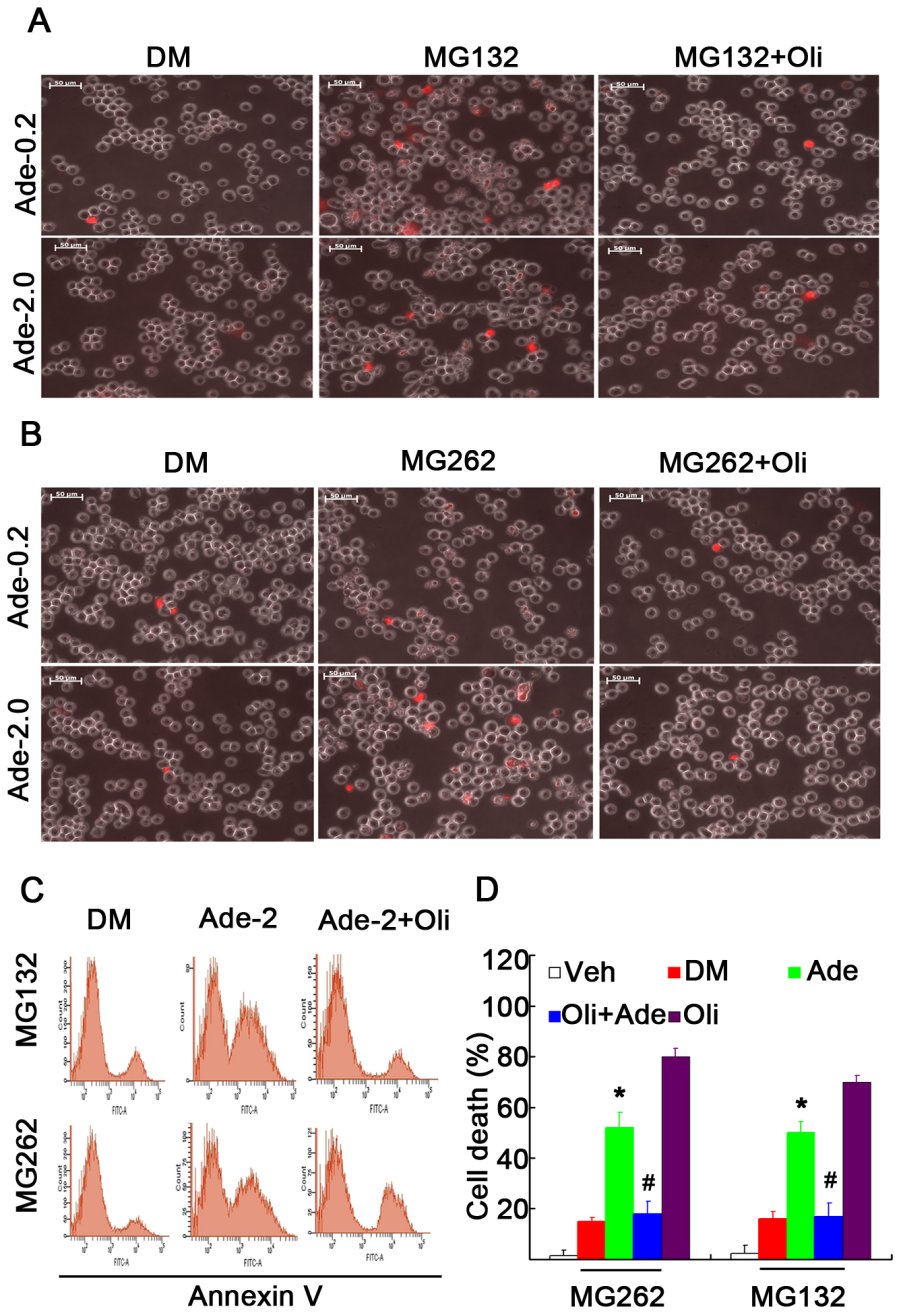
Oligomycin decreases proteasome inhibition-induced cell death in the presence of Ade. (A, B) K562 cells cultured in d-glucose-free medium were exposed to Ade (0.2 and 2 mM), MG132 (5 µM, or MG262 (1 µM) and their combinations; PI staining was dynamically recorded under a fluorescent microscope, typical images at 12 h are shown in (A) and (B). (C, D) K562 cells were treated with Oli (1 µM), MG132 (5 µM), or MG262 (1 µM) and their combinations in the absence or presence of Ade (2 mM) for 12 h; cell apoptosis was detected using Annexin V/PI staining. Typical images are shown in (C) and a summary of cell death is shown in (D). Mean +SD (n = 3). *p<0.05 *vs.* proteasome inhibitor treatment alone.

We and others have reported that intracellular concentrations of ATP determine the sensitivity to cell death induced by either proteasome inhibitors or other apoptosis inducers [15], [18], [19]. In the present study, we investigated whether Ade yielded a similar effect as ATP. We found that Ade dose-dependently enhanced proteasome inhibition-induced cell death, specifically necrosis, as detected by either Annexin V/PI staining or PI staining in live cells (Figs 5A–D). Similarly, Ade also enhanced proteasome inhibition-induced PARP cleavage, an indicator of apoptosis (Figs 5E, F). Further results showed that Oli, by efficiently inhibiting ATP generation, could mostly reverse the enhanced effects of Ade on cell death mediated by proteasome inhibition (Figs 6A–D).

## Discussion

To efficiently monitor intracellular ATP changes, several strategies were used. In normal cell cultures, ATP mainly depends on the presence of d-glucose, either by glycolysis or oxidative phosphorylation [23]. In the absence of d-glucose in the culture medium, ATP mainly comes from the non-glucose pathway which generates ATP via oxidative phosphorylation. In most cancer cells, ATP generation mostly depends on glycolysis [24]. Therefore in cancer cells cultured in the absence of d-glucose, Oli, a F_o_F_1_-ATPase inhibitor, can quickly deplete intracellular ATP (Fig. 1B). Normal cells such as thymocytes mainly rely on oxidative phosphorylation for ATP generation; Oli can deplete intracellular ATP even in the presence of d-glucose in the culture medium [19]. Ade, a precursor of ATP, can be incorporated into the compartmental pool of ATP via a specific ATP synthesis pathway and elevate the nuclear compartmental pool of ATP [25], [26]. The Ade-induced ATP production pathway is different from the glucose-mediated pathway in which Ade is rapidly metabolized via phosphorylation by Ade kinase and preferentially incorporated into ATP compared with its incorporation into AMP and ADP [25]. Ade can increase ATP generation, regardless of the presence of d-glucose, but cannot continuously increase intracellular ATP concentrations (Fig. 1A and B); and Oli cannot block Ade-mediated ATP production (Fig. 1B). DP, an inhibitor of the Ade transmembrane carrier, can efficiently block Ade transportation to the cytosol for ATP production (Fig. 4A). By using these strategies, intracellular ATP content was accurately regulated as expected.

To observe the functional effects of Ade-derived ATP on cell viability and cell death, we compared the difference with Ade treatment in low and normal ATP states. In the normal culture condition, Ade induced higher levels of ATP and decreased cell viability, either by inhibiting cell proliferation or inducing cell death, depending on different cell types (Fig. 2). The results support the notion that under normal culture conditions Ade exerts a detrimental effect on the cell, whereas in the absence of d-glucose in the culture medium, Ade increased intracellular ATP above the control level. Ade also increased cell viability above the control level. In the absence of d-glucose, Oli quickly depleted intracellular ATP and induced cell death, but the addition of Ade almost completely rescued these changes, indicating that Ade exerts its protective effects on cell viability via Ade-mediated ATP formation in low ATP states. To confirm whether Ade-induced cytotoxicity and cytoprotection was mediated by ATP formation or a receptor-mediated pathway, an Ade transmembrane inhibitor and a broad Ade receptor inhibitor were used to differentiate their effects on intracellular ATP concentration and cell viability. Blocking Ade transport to the cell by DP simultaneously blocked Ade-mediated ATP increase and cytotoxicity or cytoprotection, but not by the Ade receptor inhibitor 8-SPT (Fig. 4). These findings have been confirmed by detecting the sensitivity of Ade to proteasome inhibitors. It is well known that intracellular ATP has an important role in mediating cell death [15], [18], [19]. Consistent with ATP, Ade also determines the sensitivity to proteasome inhibition-induced cell death by increasing intracellular ATP concentrations. These results support the concept that Ade at millimolar levels is beneficial to cells under low ATP states and is detrimental to cells above normal ATP concentrations in cultured cells.

The effects of Ade in ischemic/hypoxic injury, anti-inflammation therapy, or cancer therapy have been argued for a number of years. The debates focus on the different effects of Ade receptor subtypes in different cell types [12], [13]. Tissue protection and regeneration by Ade is mediated by many different cell types and involves participation of all four Ade receptor subtypes. Fully understanding and exploiting these protective and regenerative mechanisms has significant clinical potential. Ade has also been confirmed to be effective in the treatment of inflammation and experimental cancers, and Ade receptors are becoming important molecular targets for cancer and inflammation therapy [27], [28]. In these two cases, two opposite actions exist: one is protective, the other is detrimental. It has been reported that A_2A_ and A_2B_ receptors are coupled to adenylate cyclase activity and their stimulation increases intracellular concentrations of cAMP. A_1_ and A_3_ receptor stimulation decreases cAMP concentrations and raises intracellular Ca^2+^ levels by a pathway involving phospholipase C activation [14], [29]. Other studies have shown that the stimulation of A_2A_ Ade receptors produced deleterious effects; A_3_ stimulation counteracted A_2A_-induced cell death and reduced cell proliferation. Furthermore, A_3_ stimulation ensures cell survival. These results indicated that Ade triggers a survival signal via A_3_ receptor activation and kills the cell via A_2A_ receptor activation [27]. Further studies have shown that Ade, acting at specific A_2A_ receptors, promotes wound healing *in vivo* [30], and the A_3_ Ade receptor blocks UV irradiation-induced apoptosis in mast-like cells [31]. Cells of the immune system express Ade receptors and are responsive to the modulatory effects of Ade in an inflammatory environment. The role of Ade in the control of immune and inflammatory systems has generated the potential use of Ade-receptor-based therapies in the treatment of infection, autoimmunity, ischemia, and degenerative diseases [1], [28], [32]. Despite a large body of literature, the role of A_2A_ and A_3_ in apoptosis and cell death induction remains unclear. Evidence from *in vivo* studies suggest that blocking A_2A_ achieved neuroprotection and supports the view that A_2A_ stimulation is detrimental in neurons and also in thymocytes [33], [34]. Conversely, A_2A_ receptor activation appeared to reduce ischemia-reperfusion injury in the kidney [35]. Other studies have shown that low concentrations of A_3_ receptor agonists have protective effects and, in contrast, high doses of agonists for the A_3_ receptor can induce apoptosis. The previous Ade receptor hypothesis has difficulties in explaining the different effects of Ade. In the present study, we reported a novel pathway to elucidate how and why Ade exerts double-edged effects. Even though in physiological conditions it is difficult to increase Ade as high as several millimolar levels, the findings will help us to further understand the importance of Ade, both in experimental and clinical conditions. In low ATP states, cells lack energy to perform their biological activities, therefore it is reasonable to understand that addition of Ade can increase cell viability by increasing intracellular ATP concentrations; while in normal ATP states, Ade decreased cell viability, which is consistent with previous reports [1]–[4].
